# British Institutions for the Care of the Inebriate.—III

**Published:** 1903-10-24

**Authors:** 


### HOSPITAL ADMINISTRATION.

### CONSTRUCTION AND ECONOMICS.

### BRITISH INSTITUTIONS FOR THE CARE OF THE INEBRIATE.—III.

By Our Special Medical Commissioner.

(*Continued from Page* 38.)

#### HIGH SHOT HOUSE, ST. MARGARET'S, TWICKENHAM.

Establishments for the care of inebriates, like their inmates, are of "all sorts and conditions." At present they may be divided into two great classes, the strictly private homes over which no outside controlling influence can be exercised, and the duly licensed retreats which are regularly inspected and reported on. It is most desirable that all institutions receiving inebriates should be open to Government inspection; but such is not yet. The present state of affairs is well summarised by his Majesty's Inspector in his last report:—"A retreat may be established by any person, or body of persons, who may be considered capable of conducting such an undertaking, provided that the premises to be used for the purpose are proved to be of a suitable character. A license must be obtained by the persons proposing to conduct the retreat before any patient who has signed under the Act can legally be detained therein. The County or Borough Council in whose jurisdiction the proposed retreat is situate is the authority to whom application must ultimately be made for license; but before such application is formally made, plans and all particulars concerning the retreat must be submitted to the Home Office for the approval of the Secretary of State. After the retreat is properly licensed it is subject to inspection by any person appointed by the Secretary of State for that purpose."

The statutory procedure at present regulating the granting of licenses and controlling their inspection is in many ways cumbrous and frequently causes much trouble and inconvenience, but this aspect of the case we shall have to deal with later in our series of articles.

Until compulsory inspection is made binding on all those undertaking the responsibilities connected with the care of the inebriate, medical men should exercise the greatest care and discretion in the selection of a "retreat" for their patients.

Among the private licensed institutions for the treatment of male inebriates High Shot House, situate in Crown Road at St. Margaret's, Twickenham, Middlesex, occupies a foremost place. It was established as a home for gentlemen suffering from inebriety, the morphia habit, and the abuse of drugs, in 1886, by Dr. Branthwaite, father of the present well-known expert in all matters connected with the treatment of the inebriate, H.M. Inspector Dr. Welsh Branthwaite. The present resident medical superintendent is Dr. Thelwell Pike, who has in a long general practice enjoyed peculiar opportunities for fitting himself for the direction of such an establishment. High Shot House a hundred years ago was the residence of Louis Philippe. It has of course been considerably enlarged and much altered since the days when it was the home of a royal fugitive, and now it is admirably adapted for its purpose. Its rooms are comfortable; there is a spacious dining-room and a convenient recreation-room, an excellent billiard-room with full-sized table, and reading and visitors' rooms. Plentiful arrangements have been made for amusements, such as lawn tennis, bowls and quoits, and there is a workshop and facilities for pursuing the dark art of photography. The river Thames is only six minutes' walk distant, and affords unending opportunities for boating and various forms of aquatic recreation.

In the immediate neighbourhood are many places of high historical and rich archæological interest. Kew Gardens and Richmond Park are readily accessible, and trams and electric cars run frequently to Hampton Court. The inmates are allowed a considerable amount of freedom, and pass beyond bounds in suitable charge. Life in the establishment is made as congenial as possible. There is a good library, and the leading daily papers, journals and magazines, together with professional and other periodicals are supplied. All is done to break down a sense of isolation and strengthen an independence of character.

The life of the institution has as far as we can gather been well arranged. The regulations and orders for domestic arrangement and management have been drawn with much care, and appear to work excellently. No money is allowed to be retained by the patients, and no local debts can be incurred by them without written consent. No intoxicating liquor of course may be taken or is allowed to be introduced into the premises. No drugs or medicinal preparations may be used unless prescribed by the medical superintendent. Punctuality at meals is strictly enjoined; the diet is abundant, probably too plentiful for the less energetic of the inmates. Smoking is allowed at the discretion of the doctor. Infraction of discipline renders a private patient liable to expulsion, and a patient under the Act, for any offence against the Act, may be summoned before the Justices. The various regulations and orders under which the routine of the establishment is conducted have been approved by H.M. Inspector of Retreats.

We were glad to find that much care is taken in the selection of the servants. Each has to sign an "undertaking" to continue, during residence as a servant in the house, to be a total abstainer from all intoxicating liquors and all narcotic drugs; and the same "document" reproduces the 24th section of the Inebriates Acts 1879–98 relating to offences by officers, servants, and other persons:—"If any person does any of the following things:—

"(1) Illtreats, or being an officer, servant, or other person employed in or about a retreat, wilfully neglects any habitual drunkard detained in a retreat

"(2) Induces or knowingly assists an habitual drunkard detained in a retreat to escape therefrom

"(3) Without the authority of the licensee or the medical officer of the retreat (proof whereof shall lie on him) brings into any retreat, or, without the authority of the medical officer of the retreat, except in case of urgent necessity, gives or supplies to any person detained therein, any intoxicating liquor, or sedative narcotic, or stimulant, drug or preparation,

"He shall be deemed guilty of an offence against this Act."

Inquiries directed to the manner and method of treatment elicit the information that the management of every case is based upon sound rational lines; fads and fancies are not employed but each case is submitted to individual study, and is dealt with accordingly.

Dr. Pike, we understand, is not in favour of the granting of liberty on parole during detention as he has found that such cases almost invariably relapse. It is particularly desirable for such that some "intermediate" institution or special "convalescent" home should be found and that a wise "after care" should for long be exercised over.

We ascertained that the following are the terms for admission:—Entrance fee, £1 1s. A minimum term of thirteen weeks' board and residence in advance from £2 12s. 6d. to £5 5s. per week, according to bedroom accommodation. The following are charged as extras:—Laundry, private sitting room, meals and fire in bedroom, billiards and medicine. No allowance can be demanded for an unexpired period in case of determination of contract. Special arrangements and terms may be made for permanent residence and also for short periods of less than three months. The nearest station is St. Margaret's, on the London and South-Western Railway.

(*To be continued.*)

